# Genome-Wide Profiling Reveals HPV Integration Pattern and Activated Carcinogenic Pathways in Penile Squamous Cell Carcinoma

**DOI:** 10.3390/cancers13236104

**Published:** 2021-12-03

**Authors:** Kang-Bo Huang, Sheng-Jie Guo, Yong-Hong Li, Xin-Ke Zhang, Dong Chen, Philippe E. Spiess, Zai-Shang Li, Chuang-Zhong Deng, Jie-Ping Chen, Qiang-Hua Zhou, Zheng Hu, Xin Ma, Jie-Tian Jin, Yun Cao, Jun-Hang Luo, Xiao-Bin Wang, Fang-Jian Zhou, Ran-Yi Liu, Hui Han

**Affiliations:** 1 State Key Laboratory of Oncology in South China, Collaborative Innovation Center for Cancer Medicine, Sun Yat-sen University Cancer Center, Guangzhou 510060, China; huangkb3@mail2.sysu.edu.cn (K.-B.H.); guoshj@sysucc.org.cn (S.-J.G.); liyongh@sysucc.org.cn (Y.-H.L.); zhangxk@sysucc.org.cn (X.-K.Z.); chendong1@sysucc.org.cn (D.C.); dengchzh@sysucc.org.cn (C.-Z.D.); chenjp@sysucc.org.cn (J.-P.C.); jinjt@sysucc.org.cn (J.-T.J.); caoyun@sysucc.org.cn (Y.C.); zhoufj@sysucc.org.cn (F.-J.Z.); 2 Department of Urology, Sun Yat-sen University Cancer Center, Guangzhou 510060, China; 3 Department of Pathology, Sun Yat-sen University Cancer Center, Guangzhou 510060, China; 4 H. Lee Moffitt Cancer Center and Research Institute, Tampa, FL 33612, USA; philippe.spiess@moffitt.org; 5 Department of Urology, Shenzhen People’s Hospital, The First Affiliated Hospital of Southern University of Science and Technology, Shenzhen 518020, China; dean2011@163.com; 6 Department of Orthopedics, Sun Yat-sen University Cancer Center, Guangzhou 510060, China; 7 Department of Gynecology, Sun Yat-sen University Cancer Center, Guangzhou 510060, China; 8 Department of Urology, Sun Yat-sen Memorial Hospital, Guangzhou 510120, China; zhouqhsysu@163.com; 9 Department of Obstetrics and Gynecology, Precision Medicine Institute, The First Affiliated Hospital of Sun Yat-sen University, Guangzhou 510080, China; huzheng1998@163.com; 10 Department of Urology, The General Hospital of the People’s Liberation Army, Beijing 100853, China; urologist@foxmail.com; 11 Department of Urology, The First Affiliated Hospital of Sun Yat-sen University, Guangzhou 510080, China; luojunh@mail.sysu.edu.cn; 12 MyGenostics Inc., Beijing 101318, China; wangxiaobin@mygeno.cn

**Keywords:** penile squamous cell carcinoma, human papillomavirus, E2, MAPK signaling pathway, CADM2

## Abstract

**Simple Summary:**

Penile squamous cell carcinoma (PSCC) has been regarded as an HPV-related cancer for a long time. However, the integration pattern and carcinogenic pathways of HPV in PSCC remain unclear. The results of this study provide insights into the HPV-related carcinogenic mechanism in PSCC, which may be less prone to involvement in the traditional *E6*/*E7* carcinogenic process, and are characterized by effects on the host genome, which result in the inactivation of tumor suppressors (*CADM2*, etc.) and the activation of oncogenes (*KLF5*, etc.), thus activating oncogenic signaling pathways (MAPK, JAK/STAT, etc.). This study could enhance our understanding of HPV integration and pave the way for subsequent HPV studies in PSCC.

**Abstract:**

Human papillomavirus (HPV) is a significant etiologic driver of penile squamous cell carcinoma (PSCC). The integration pattern of HPV and its carcinogenic mechanism in PSCC remain largely unclear. We retrospectively reviewed 108 PSCC cases who received surgery between 2008 and 2017. Using high-throughput viral integration detection, we identified 35 HPV-integrated PSCCs. Unlike cervical cancer, the HPV *E2* oncogene was not prone to involvement in integration. Eleven of the 35 (31.4%) HPV-integrated PSCCs harbored intact HPV *E2*; these tumors had lower HPV *E6* and *E7* expression and higher expression of p53 and pRb proteins than those with disrupted *E2* did (*p* < 0.001 and *p* = 0.024). Integration breakpoints are preferentially distributed in or near host genes, including previously reported hotspots (*KLF5*, etc.) and newly identified hotspots (*CADM2*, etc.), which are mainly involved in oncogenic signaling pathways (MAPK, JAK/STAT, etc.). Regarding the phosphorylation levels of JNK, p38 was higher in HPV-positive tumors with MAPK-associated integration than those in HPV-positive tumors with other integration and those in HPV-negative tumors. In vitro, *KLF5* knockdown inhibited proliferation and invasion of PSCC cells, while silencing *CADM2* promoted migration and invasion. In conclusion, this study enhances our understanding of HPV-induced carcinogenesis in PSCC, which may not only rely on the *E6*/*E7* oncogenes, but mat also affect the expression of critical genes and thus activate oncogenic pathways.

## 1. Introduction

Penile squamous cell carcinoma (PSCC) is a rare malignancy worldwide, but its incidence is much higher in developing areas such as Africa, South America, and Southeast Asia. Human papillomavirus (HPV) infection is a significant etiologic driver of PSCC [1]. In most instances, the HPV genome is detected as integrated rather than episomal in tumors [2]. The integration of the HPV genome into the host genome has an important role in HPV-induced carcinogenesis. According to studies of other HPV-related cancers, the most common carcinogenic process is *E6*/*E7*-induced carcinogenesis triggered by disruption of the HPV *E2* oncogene: The disruption of *E2* results in a loss of negative feedback control of the expression of the *E6* and *E7* viral oncoproteins that are involved in the inactivation of p53 and pRb proteins [3,4,5]. Besides traditional *E6*/*E7*-induced carcinogenesis, in some cases, HPV integration also induces carcinogenesis through directly activating oncogenes or inactivating tumor suppressors in the human genome [6,7]. In other HPV-related cancers, thousands of HPV integration sites in the human genome have been identified, and many distribute near the known cancer driver genes [8,9]. However, the carcinogenic mechanism of HPV integration in PSCC remains largely unknown.

The integration pattern and characteristics of HPV in the PSCC genome are also unclear. Several studies have indicated that HPV-positive PSCC harbors a different genomic profile [10,11,12,13] and specific epigenetic alterations [12] when compared to HPV-negative PSCC or normal tissue. For instance, HPV-positive PSCC often harbors an *APOBEC* mutation [11]; copy number variations (CNVs) of 19 genomic regions are commonly detected in HPV-positive tumors [10]; HPV-positive tumors are characterized by the widespread loss of DNA methylation [12]. However, these studies mainly indirectly investigated HPV integration by demonstrating the molecular characteristics of HPV-positive tumors, instead of directly investigating HPV genome integration in the PSCC genome. Thus, we aimed to determine the HPV integration landscape in the PSCC genome using high-throughput viral integration detection (HIVID), and to clarify the pattern and impact of HPV integration in PSCC.

## 2. Materials and Methods

### 2.1. PSCC Patients and Samples

**Figure 1:**
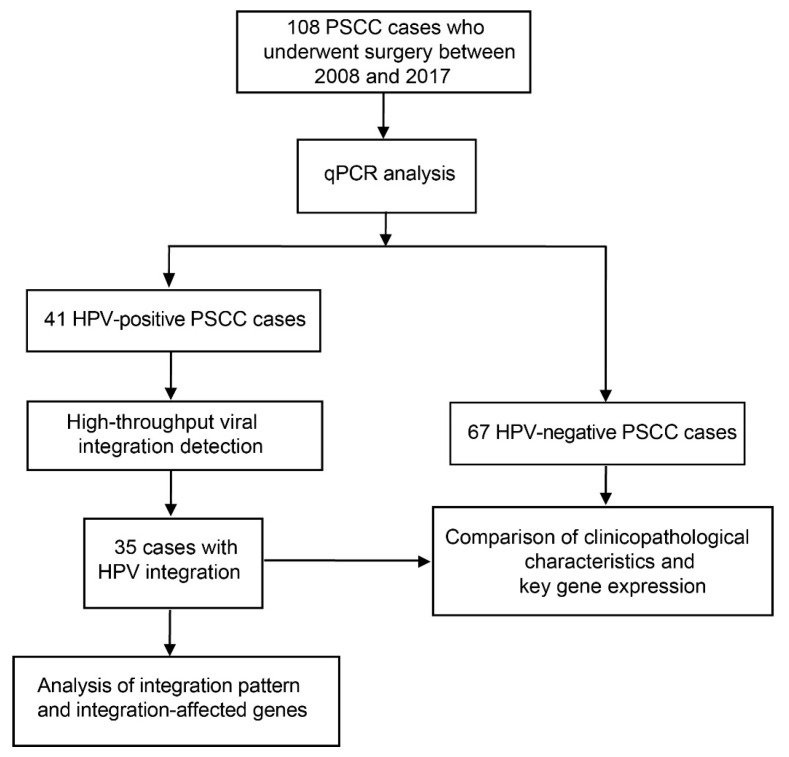
Study design. PSCC, penile squamous cell carcinoma; qPCR, quantitative polymerase chain reaction.

In this study, we used tumor specimens from 108 PSCC patients who underwent surgical intervention at the Sun Yat-sen University Cancer Center between 2008 and 2017. The inclusion criteria were as follows: (I) Histologically confirmed PSCC; (II) no anti-cancer treatments received before surgery; (III) no history or concurrence of other malignant tumors; (IV) complete clinicopathological data. Histologic subtypes of PSCC were reviewed by two pathologists according to the fourth edition of the World Health Organization’s classification [14]. Tumor specimens were collected during surgery and were frozen at −80 °C. All of the specimens were managed carefully to avoid cross-contamination. Forty-one of them were identified as HPV-positive by quantitative polymerase chain reaction (qPCR) (harbored HPV DNA, which were positive in HPV *E6* and *E7* expression); 35 of the 41 PSCCs were verified to harbor HPV integration using HIVID analysis and further analysis of HPV integration was performed. Moreover, 67 HPV-negative cases were used to compare the clinicopathological characteristics and the expression of key genes with 35 HPV-integrated cases. The study design is shown in Figure 1. Detailed information of the 35 HPV-integrated PSCCs and 67 HPV-negative PSCCs is shown in Table 1.

### 2.2. Library Preparation and HIVID

HPV^+^ PSCC samples were subjected to library preparation as previously reported (described in Supplementary Figure S1) [15]. Briefly, 3–5 μg of genomic DNA was cleaved into approximately 150 bp DNA fragments using a Covaris S2 Focused Ultrasonicator (Covaris, Inc., Woburn, MA, USA). After purification, the fragments were end-blunted, A-tailed, adaptor-ligated, and amplified by 15 cycles of PCR. The concentration of the library was quantified by a Nanodrop 2000 system (Thermo Fisher Scientific, Indianapolis, IN, USA), and the fragment size was resolved by 1% agarose gel electrophoresis.

Targeted DNA fragments were captured from the prepared libraries by hybridizing with biotin-labeled probes (MyGenostics, Baltimore, MD, USA) covering the full-length HPV genomes of 18 subtypes (Supplementary Table S1), namely, HPV6, HPV11, HPV16, HPV18, HPV31, HPV33, HPV35, HPV39, HPV45, HPV51, HPV52, HPV56, HPV58, HPV59, HPV66, HPV68, HPV69, and HPV82, and then were washed to remove non-target fragments. The captured DNA fragments were subjected to sequencing and paired-end high-throughput sequencing using an Illumina HiSeq 2500 sequencer (Illumina Inc., San Diego, CA, USA), and the raw data were deposited into the National Center for Biotechnology Information (NCBI; SRA accession number: SRR9112949-SRR9112989). The sequencing coverage and quality statistics of each sample are summarized in Supplementary Table S2.

### 2.3. Bioinformatic Analysis for HPV Integration

Adaptor, low-quality, and short sequence (less than 40 bp) data were first removed to obtain clean data, which were then mapped to 314 genomes of 32 HPV subtypes (Supplementary Table S3) using Burrows–Wheeler Aligner (BWA) [16]. The basic information on the HPV subtype was output according to the mapping results meeting the criteria of (i) an average sequencing depth ≥10 and (ii) more than 45% of the data showing >4× coverage. Then, the data were mapped to the human genome (NCBI, hg19) and HPV-integrated reference genomes were selected according to basic subtype information using BWA MEM to obtain mapped bam results. The reads of each detected HPV type in PSCC samples are listed in Supplementary Table S4.

Next, the initial integration sites were obtained by SVdetect analysis [17], high-confident HPV integration sites were obtained by CREST analysis [18], the consensus sequence near the integration site was collated, while the SVdetect analysis results without integration sites detected by CREST were supplemented with low-confidence results. Chimeric paired-end reads (partially aligned to the human genome and partially to the HPV genome) were defined as HPV integration and the joint positions in the human and HPV sequences were identified as integration breakpoints (Supplementary Figure S1) [9,15].

Subsequently, the genomic regions/elements and genes involved in HPV integration were analyzed. High-confidence results were annotated using ANNOVAR [19], referring to the repetitive region in the hg19 build. Regions with integration breakpoints and genome-unstable elements containing the breakpoints or those that were <200 bp from these regions were included. The observed count in a certain area was compared with the expected value that was calculated as a randomized distribution in the genome. The annotated genes that were <500 kb from the high-confidence breakpoints were regarded as possibly affected genes, as previously described [9], and a gene that appeared repetitively in the same sample was counted once. Gene family classification and Kyoto Encyclopedia of Genes and Genomes (KEGG) pathway analysis were performed using Gene Set Enrichment Analysis [20,21], and a significant enrichment was identified when the false discovery rate (FDR) *q*-value was <0.05.

### 2.4. Immunohistochemistry

After routine deparaffinization, rehydration, antigen retrieval, and endogenous peroxidase inactivation, FFPE specimen slides (4.0 μm-thick) were subjected to immunohistochemistry (IHC) staining using primary antibodies against phospho-(p-)JNK (1:100; 81E11, Cell Signaling Technology, Inc. (CST), Danvers, MA, USA), p-ERK (1:100; D13.14.4E, CST), p-p38 (1:100; D3F9, CST), p-STAT3 (1:100; D3A7, CST), β-catenin (1:100; D10A8, CST), CEP19 (1:200; HPA047614, Sigma-Aldrich), NRROS (Sigma-Aldrich, HPA031586, 1:50), CADM2 (1:50; #46390, SAB biotech, Nanjing, China) or KLF5 (1:100; HPA040398, Sigma-Aldrich), p53 (1:100, 7F5, CST), pRb (1:500, 4H1, CST), and the Dako REAL™ EnVision™ Detection System (Dako, Glostrup, Denmark) according to the manufacturer’s instructions. To score the IHC staining, 3–5 (depending on the size of the tumor area) independent microscopic fields (100×) of each tumor specimen were examined in terms of the integrated optical density (IOD) via Image Pro Plus 6.0 (Media Cybernetics, Silver Spring, USA) [22]. The staining positivity or negativity was judged according to the cut-off value of IOD determined by X-tile (New Haven, CT, USA) for optimal survival separation.

### 2.5. Cell Culture, siRNA/Plasmid Transfection, and Cell Proliferation Assay

Two PSCC cell lines previously established by our group, Penl2 (RRID: CVCL_VF95) and 149RCa (RRID: CVCL_VF96) [23], were cultured in DMEM (Gibco, Waltham, MA, USA) supplemented with 10% FBS (Thermo Fisher Scientific, Waltham, MA, USA). All human cell lines were authenticated using short tandem repeat profiling within the last three years and all experiments were performed with mycoplasma-free cells. Specific siRNAs (si-NC was used as negative control siRNA) (Supplementary Table S5) or CAMD2 cDNA (blank vector was used as negative control, from GenePharma: Suzhou, Jiangsu, China) were transiently transfected into PSCC cells to knock down or overexpress the indicated gene using Lipofectamine 3000 (Invitrogen, Carlsbad, CA, USA) according to the manufacturer’s guidelines. Then cells were subjected to cell proliferation and migration/invasion assays. For cell proliferation assay, PSCC cells (3000/well) were seeded in 96-well plates and cultured for indicated times, and cell proliferation was analyzed using a cell viability assay by a cell counting kit-8 (Dojindo, Tokyo, Japan) and represented as the optical density at 450 nm (OD_450nm_) [24].

### 2.6. Migration and Invasion Assay

The migration ability of PSCC cells was assessed using a wound healing assay [23,25]. Briefly, cells were seeded in 6-well plates and grown to confluence, when the cell monolayer was generating a straight scratch using 200 μL tip and followed by a gentle wash. The scratched monolayer was incubated with fresh serum-free medium and imaged at 0 h and 12 h after incubation. The migration ability was presented as the percentage of wound healing.

The invasion ability of PSCC cells was evaluated using a Transwell assay as described previously [24,26]. Briefly, 2 × 10^5^ 149RCa cells or 1 × 10^5^ Penl2 cells were seeded into Boyden chambers (8 µm pore) with Matrigel coating (BD Biosciences, San Jose, CA, USA). After 24 or 36 h of incubation, the cells on the lower surface of the filter were fixed, stained, and counted.

### 2.7. RNA Extraction and qPCR

Total RNA was extracted from PSCC cells or RNA later-preserved tissues using Trizol reagent (Invitrogen, USA), and then reverse-transcribed into cDNA using HiScript II (Vazyme, China). qPCR assay was performed using SYBR^®^ Premix Ex Taq^TM^ (TaKaRa) with specific primers (Supplementary Table S6). The relative mRNA levels were presented as the 2^−ΔCt^ (GAPDH was used as an internal control) after being normalized to the control (negative control or corresponding normal tissue).

### 2.8. Statistical Analysis

All statistical analyses were performed using SPSS 22.0 (IBM Corp., Armonk, NY, USA). The *χ*^2^ test, Fisher’s exact test, and Pearson’s correlation analysis were employed for correlation analysis, the Mann–Whitney and Kruskal–Wallis tests were used for rank-sum testing, and the Kaplan–Meier method with the log-rank test was used for survival analysis. Disease-specific survival was defined as the time from surgery to either death caused by disease or last follow-up. The difference between two or more groups was analyzed by Student’s *t*-test or analysis of variance (ANOVA). Statistical significance was set at *p* < 0.05.

## 3. Results

### 3.1. HPV Integration Status and HPV Subtypes in PSCC

We performed an HIVID assay in 41 HPV-positive PSCC previously identified by qPCR (Figure 1). HIVID detected HPV DNA in 38 of 41 cases, including 11 HPV subtypes: HPV16 (33/38, 86.8%), HPV51 (4/38, 10.5%), HPV33 (3/38, 7.9%), HPV56 (2/38, 5.3%), and HPV6, 18, 44, 58, 62, 66, and 68 (1/38, 2.6%) (Supplementary Table S7). Most samples (31/38, 81.6%) were infected with a single subtype of HPV, whereas 18.4% (7/38) were infected with multiple subtypes.

Among the 38 HPV DNA-positive cases detected by HIVID, 35 of them (92.1%) were observed HPV integration, while the rest three tumors only harbored episomal HPV DNA. In total, 2252 HPV integration breakpoints were identified in 35 PSCC specimens (median breakpoints, 14.0; 95% confidence interval, 0.81 to 127.9) (Supplementary Tables S8 and S9). Among the 11 aforementioned HPV subtypes, only five high-risk subtypes—HPV16 (33/35, 94.3%), HPV33 (2/35, 5.7%), and HPV 51, 58, and 68 (1/35, 2.9%)—showed integration into host genome in tumors, while the other six subtypes (0/35, 0%) showed no integration (Supplementary Table S8 and Supplementary Figure S2). Comparing 35 HPV-integrated tumors with 67 HPV-negative tumors, we found that tumors with HPV-related histologic subtypes were more prone to be involved in integration compared with those with non-HPV-related histologic subtypes (*p* < 0.001) (Table 1).

### 3.2. HPV Integration Pattern with Respect to the Human PSCC Genome

**Figure 2:**
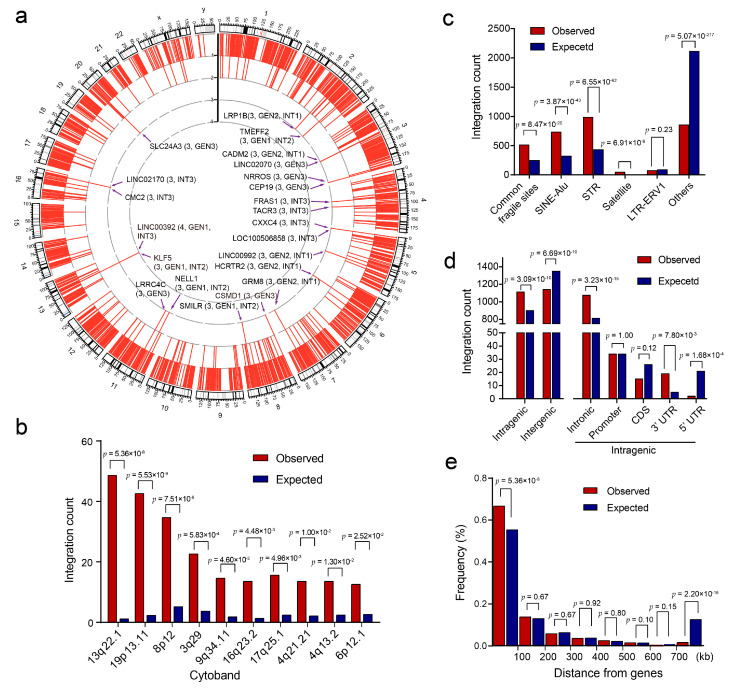
HPV integration characteristics in the penile squamous cell cancer genome. (**a**) Distribution of integration breakpoints in the human genome. In the outer circle, each bar represents the location of HPV integration in human chromosomes. In the inner circle, each red bar represents an HPV integration event and the bar length indicates the sample count (histogram axis units denote the sample count). Integration hotspots, within or <500 kb from genes, are marked specifically. (**b**–**e**) Comparison of the observed (**red**) and expected (**blue**) numbers of breakpoints in the top 10 integrated cytobands (**b**), genomic instability-related elements (**c**), intra-/intergenic regions of human annotated genes (**d**), or different distances from genes (**e**). STR, short tandem repeat. *p*-Values were calculated by the chi-squared test.

We deciphered the pattern of HPV integration in the human genome and found that although the integration event distributed in all chromosomes in the human genome (Figure 2a), they were mainly clustered in chromosomes 8, 13, and 19 and were less prone to occur in chromosome 22 and the sex chromosomes (Supplementary Figure S3). Specifically, integration breakpoints were enriched in cytobands 13q22.1, 19p13.11, 8p12, and 3q29 (Figure 2b). Furthermore, the breakpoints were enriched in or around genome instability elements (Figure 2c), such as short tandem repeats (*p* = 6.6 × 10^−62^), common fragile sites (*p* = 8.5 × 10^−25^), SINE-Alu (*p* = 3.9 × 10^−43^), and satellites (*p* = 6.9 × 10^−9^), but were relatively scarce in areas without those elements (*p* = 5.1 × 10^−217^).

As to the gene level, we found a significant enrichment tendency of HPV integration toward host genes in PSCC. The integration breakpoints were significantly enriched in intragenic regions (*p* = 3.1 × 10^−10^), but not in intergenic regions (*p* = 6.7 × 10^−10^) (Figure 2d). In intragenic regions, breakpoints were prone to occur in intronic (*p* = 3.2 × 10^−5^) and 3′ untranslated regions (*p* = 7.8 × 10^−3^), but not in 5′ untranslated regions (*p* = 1.7 × 10^−4^); in intergenic regions, breakpoints were clustered in regions that were <100 kb from genes (67%, *p* = 5.4 × 10^−8^), but were scarcer in regions >700 kb from genes (*p* = 2.2 × 10^−16^) (Figure 2e).

### 3.3. HPV E2 Disruption by Viral DNA Integration Is Not a Necessary Event in HPV-Induced Penile Carcinogenesis

**Figure 3:**
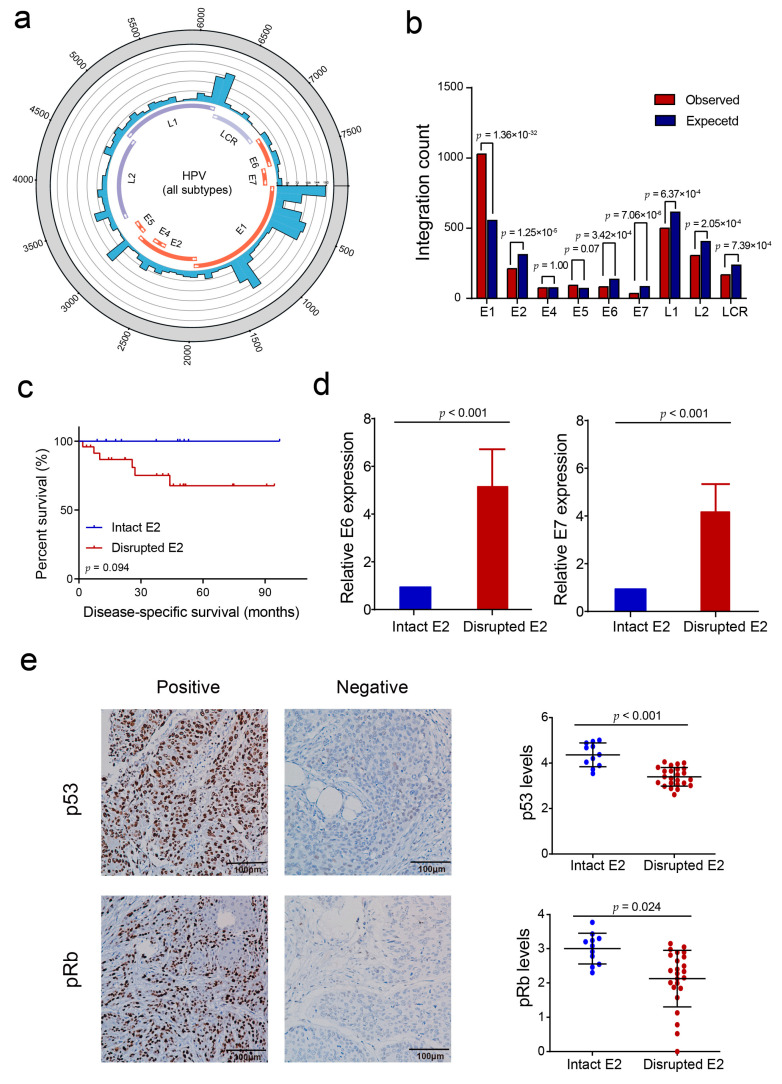
Integration profile in the HPV genome. (**a**) Distribution of integration breakpoints in all subtypes of the HPV genome. (**b**) HPV integration breakpoints in different viral genome regions; the observed (**red**) and expected (**blue**) numbers of breakpoints were compared. *p*-Values were calculated by the chi-squared test. (**c**) Kaplan–Meier survival analysis for disease-specific survival in 11 patients with intact HPV *E2* and 24 patients with disrupted HPV *E2*. (**d**) Relative expression of HPV *E6* and *E7* in tumors with intact or disrupted HPV *E2* by qPCR. (**e**) The expression of p53 and pRb in PSCC. (**left**) Representative immunohistochemistry images (100×) for p53 and pRb. (**right**) Comparison of expression levels, represented by the log_10_ (integrated optical density + 1) value, in specimens with disrupted and intact *E2*. The significance of the difference was analyzed by ANOVA. Statistical significance was set as *p* < 0.05.

Traditional *E6*/*E7* carcinogenic process often triggered by HPV *E2* disruption. The integration of *E2* causes the disruption of *E2*, thus increasing the level of HPV E6/E7 protein and decreasing the level of p53 and pRb [3,4,5]. Here, we depicted the integration pattern with respect to the HPV genome in PSCC and found that although every HPV oncogene could be involved in integration (Figure 3a), HPV *E2* was significantly less prone to be involved in HPV integration than expected (*p* = 1.25 × 10^−5^) (Figure 3b), which is different from the result in cervical cancer [9]. In total, 11/35 PSCC cases harbored an intact HPV *E2* oncogene, and among the remaining 24 *E2*-disrupted tumors, 50% (12/24) of them harbored <3 HPV *E2* integration events. Although the number of cases is relatively small, patients with intact *E2* had higher disease-specific survival rates than those with disrupted *E2* (*p* = 0.094) (Figure 3c). In tumors with intact HPV *E2*, *E6* and *E7* mRNA expression was significantly lower than those in *E2*-disrupted tumors (*p* < 0.001) (Figure 3d). Moreover, we analyzed the expression of p53 and pRb by IHC staining, and found that p53 and pRb expression in tumors with intact *E2* were significantly higher than those in *E2*-disrupted tumors (*p* < 0.001 and *p* = 0.024, respectively) (Figure 3e). Other HPV regions showed different tendencies with respect to integration. HPV *E1* (*p* = 1.36 × 10^−32^) was more prone to involve in integration, while several other regions, such as *E6* (*p* = 3.42 × 10^−4^), *E7* (*p* = 7.06 × 10^−6^), and the LCR region (*p* = 7.39 × 10^−4^) were less prone to involvement in integration (Figure 3b). The whole HPV16 genome were involved in integration, while only several regions of other subtypes’ genomes experienced integration events. Comparing HPV16 and other subtypes, we found several similarities, such as the numerous breakpoints at the beginning of LCR, the beginning and middle of *E1*, and some events in *E5* and the beginning of *L2* (Supplementary Figure S4).

### 3.4. HPV Integration May Affect the MAPK and JAK/STAT Signaling Pathways in PSCC

**Figure 4:**
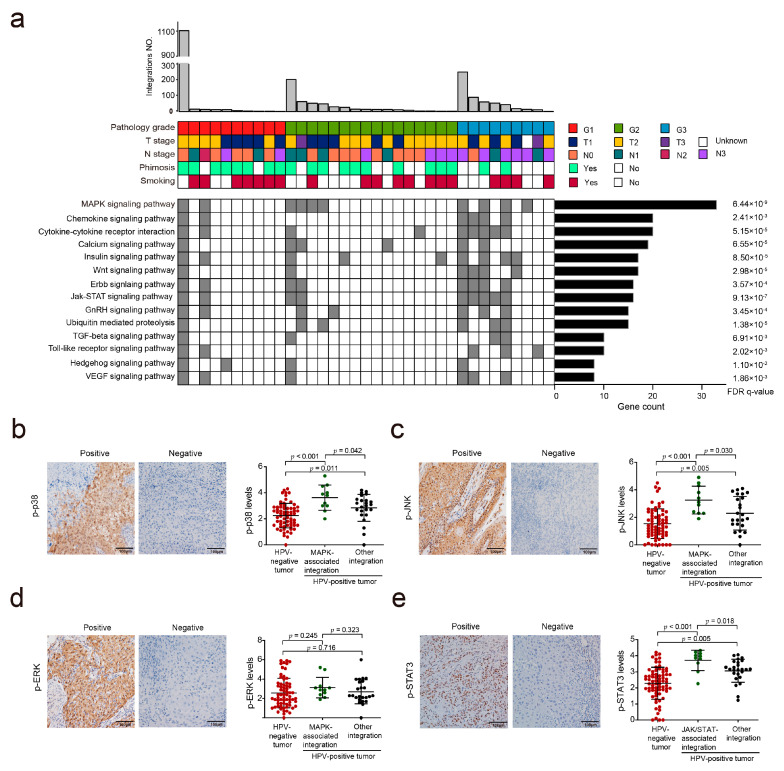
HPV-associated clinicopathological characteristics and signaling pathways in PSCCs. (**a**) The clinical annotations and main affected pathways of HPV integration in 35 PSCC cases. Each vertical track represents an individual. The data are sorted by the number of HPV integrations (**top**), pathology grade, T stage, N stage, phimosis, smoking (**middle**), and the main affected pathways, including canonical cancer-related pathways (MAPK, Wnt, Erbb, TGF-β, Hedgehog, etc.) and inflammation-related pathways (**bottom**). FDR *q*-values were generated by Gene Set Enrichment Analysis. (**b**–**d**) The expressions of p-p38 (**b**), p-JNK (**c**), and p-ERK (**d**) were compared between HPV-negative tumors, HPV-positive tumors with MAPK-associated integration, and HPV-positive tumors with other integration. Left, representative images (100×); right, bar chart for the levels of these factors in three groups represented by the log_10_ (integrated optical density + 1) value (ANOVA). (**e**) The expressions of p-STAT3 were compared between HPV-negative tumors, HPV-positive tumors with JAK/STAT-associated integration, and HPV-positive tumors with other integration. Statistical significance was set as *p* < 0.05.

As HPV integration could also induce carcinogenesis by activating oncogenes or inactivating tumor suppressors in the human genome, to investigate which gene and signaling pathway play a key role in HPV-induced PSCC, we identified 2277 genes that were likely affected by HPV integration (Supplementary Table S10). These genes included 32 oncogenes, 31 encoding cytokines and growth factors, 136 encoding transcription factors, and 60 encoding protein kinases (Supplementary Table S11 and Supplementary Figure S5). KEGG pathway analysis revealed that these genes were mainly enriched in some canonical cancer-related pathways (MAPK, Wnt, and JAK/STAT) and inflammation-related pathways (chemokine and cytokine–cytokine receptor interaction) (Figure 4a and Supplementary Table S12). This enrichment was more significant in specimens with a high histologic grade (G_3_) than in those with grade G_1–2_ (MAPK, *p* = 0.044; WNT, *p* = 0.012; JAK/STAT, *p* = 0.038; chemokine, *p* = 0.012; cytokine–cytokine receptor, *p* = 0.017).

We first analyzed the activation of the MAPK pathway, the most enriched pathway (*p* = 6.44 × 10^−^^9^; Figure 4a), in PSCC specimens by examining the phosphorylation levels of ERK, p38, and JNK, three critical kinases in the MAPK pathway. We divided PSCCs into HPV-positive tumors with MAPK-associated integration, HPV-positive tumors with other integration, and HPV-negative tumors. The results showed that HPV-positive tumors with MAPK-associated integration harbored increased levels of p-p38 and p-JNK, but not of p-ERK, compared to HPV-positive tumors with other integration (*p* = 0.042, 0.030, and 0.323, respectively) and HPV-negative tumors (*p* < 0.001, *p* < 0.001, and *p* = 0.245, respectively) (Figure 4b–d). Next, we analyzed the activation of the JAK/STAT and Wnt signaling pathways by examining the nuclear localization of p-STAT3 and β-catenin, respectively. HPV-positive tumors with JAK/STAT-associated integration harbored increased nuclear p-STAT3 levels compared to HPV-positive tumors with other integration (*p* = 0.018) and HPV-negative tumors (*p* < 0.001) (Figure 4e). However, there were no such differences when examining the nuclear β-catenin levels on PSCCs to analyze the activation of Wnt signaling pathway (*p* = 0.463 and *p* = 0.784, respectively) (Supplementary Figure S6). Taken together, these results indicate that HPV integration is correlated with the activation of the MAPK and JAK/STAT signaling pathway.

### 3.5. Hotspot Genes Affected by HPV Integration in PSCC

**Figure 5:**
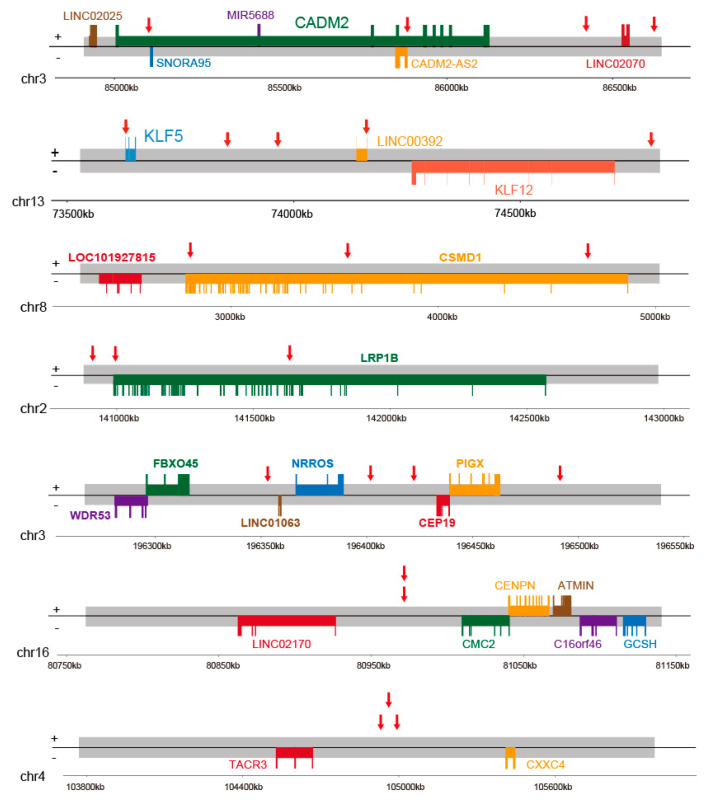
Mapping HPV integration hotspots in 35 samples. Representative integration-affected genes (*CADM2*–*LINC02070*, *KLF5*–*LINC00392*–*KLF12*, *CSMD1*, *LRP1B*, *NRROS*–*CEP19*, *LINC02170*–*CMC2*, and *TACR3*–*CXXC4*) were mapped according to the human hg19 reference genome. Red arrow, location in a given sample; Chr, chromosome; boxes, genes; bulges, exons.

Among all potential genes affected by HPV integration in PSCC, some genes that occurred in multiple cases were termed hotspot genes: 24 genes with ≥3 events and 216 genes with ≥2 events (Figure 2a and Supplementary Table S10). Some hotspot genes (e.g., *KLF5*, *LRP1B*, and *KLF12*) have previously been described in cervical cancer [9], and others (e.g., *CADM2*, *CEP19*, *CSMD1*, and *NRROS*) are reported for the first time in the present study. Moreover, some hotspot genes were located in closer proximity, such as *KLF5*–*LINC00392*–*KLF12*, *CADM2*–*LINC02070*, *TACR3*–*CXXC4*, *NRROS*–*CEP19*, and *LINC02170*–*CMC2*, which may have been affected by shared integrations (Figure 5).

**Figure 6:**
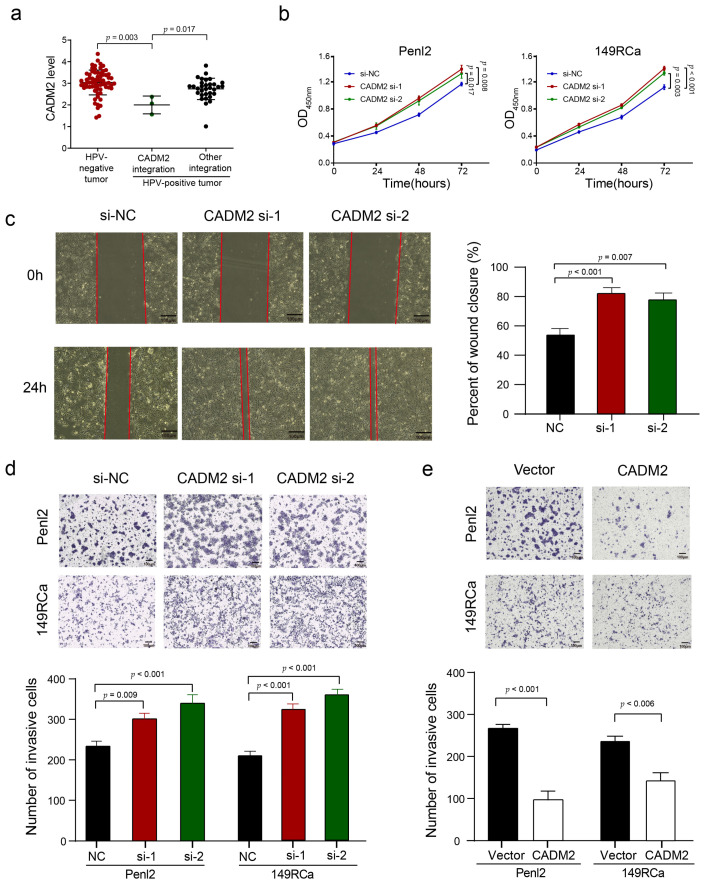
The integration hotspot gene *CADM2* affects PSCC’s proliferation and invasion ability. (**a**) Comparison of the CADM2 expression level between HPV-negative tumor, HPV-positive tumor with *CADM2* integration, and HPV-positive tumor with other integration. The expression of three groups, represented by the log_10_ (IOD + 1) value, were compared by ANOVA. (**b**) The CCK-8 assay showed the proliferation ability of *CADM2*-knockdown PSCC cells. (**c**) The wound healing assay exhibited the migration ability of *CADM2*-knockdown Penl2 cell line. Scale bar, 100 μm. (**d**,**e**) Transwell assay that examined the invasion ability of Penl2 (**d**) and 149RCa € after knockdown or overexpression CADM2. Scale bar, 100 μm. Data are shown as mean ± SD, and the *p*-value was calculated via Student’s *t*-test.

**Figure 7:**
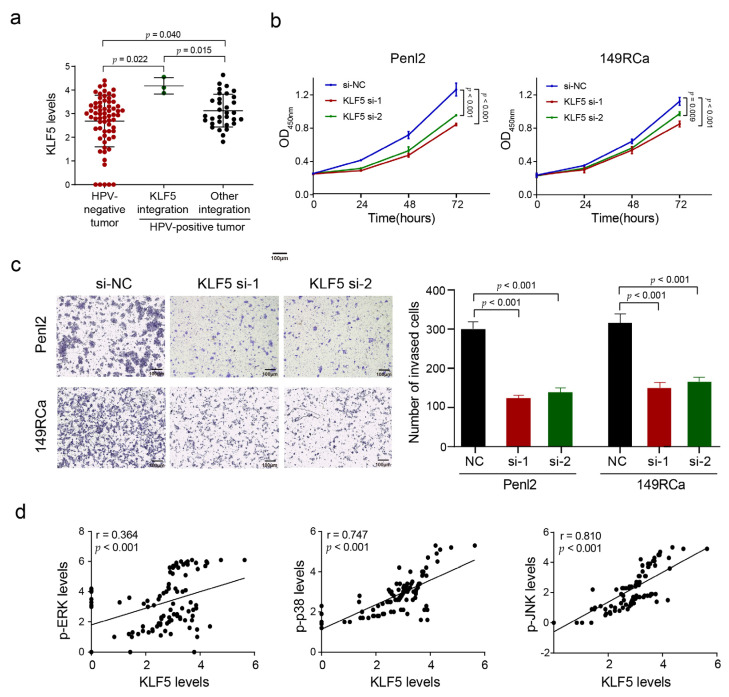
The integration hotspot gene *KLF5* promotes PSCC proliferation and invasion. (**a**) Comparison of the KLF5 expression level among HPV-negative tumors, HPV-positive tumors with *KLF5* integration, and HPV-positive tumors with other integration. The expression levels were represented by the log_10_ (IOD + 1) value. The significance of the difference between three groups was analyzed by ANOVA. (**b**) The CCK-8 assay showed the effect of *KLF5* knockdown on the proliferation in two PSCC cell lines. The *p*-value was calculated by Student’s *t*-test. (**c**) Transwell assay exhibited the invasion ability of *KLF5*-knockdown PSCC cell lines. The difference between the NC group and *KLF5*-knockdown group was compared by Student’s *t*-test. Scale bar, 100 μm. (**d**) The correlation between the expression level of p-JNK, p-ERK, p-p38, and KLF5. The expression levels were represented by the log_10_ (IOD + 1) value. Data are shown as mean ± SD, *p* value was calculated via Pearson correlation analysis.

To determine whether HPV integration affected the expression of host genes in PSCC, we chose several hotspot genes (*CADM2*, *CEP19*, *KLF5*, and *NRROS*) to examine their expression in PSCC by IHC staining (Supplementary Figure S7A–D). Compared to HPV-negative tumors and HPV-positive tumors with other integration, CADM2 expression was significantly downregulated in HPV-positive tumors with *CADM2* integration (*p* = 0.003 and 0.017, respectively) (Figure 6a), KLF5 levels were upregulated in HPV-positive tumors with *KLF5* integration than those in HPV-negative tumors and HPV-positive tumors with other integration (*p* = 0.022 and 0.015, respectively) (Figure 7a). However, the expression levels of CEP19 and NRROS showed no such difference (Supplementary Figure S7E,F).

### 3.6. CADM2 Inhibited the Proliferation and Invasion of PSCC Cells In Vitro

CADM2, a member of the cell adhesion molecule family, was involved in three events of HPV integration: Two occurred in the intronic region of *CADM2*, and another occurred in the downstream region. We report *CADM2* as an HPV-integration hotspot gene in this study. We preliminarily investigated the in vitro function of CADM2, and found that knock down of *CADM2* (Supplementary Figure S8A) significantly promoted the proliferation of PSCC cells (all *p* < 0.01; Figure 6b), and enhanced the migration ability of Penl2 (all *p* < 0.01; Figure 6c); meanwhile, the invasion ability of Penl2 and 149RCa cells (all *p* < 0.01; Figure 6d) and the overexpression of CADM2 (Supplementary Figure S8B) diminished the invasion ability of this two PSCC cell lines (all *p* < 0.01; Figure 6e). These data suggest that HPV integration may inhibit CADM2 expression and thus impair the tumor-suppressive role of CADM2 in PSCC.

### 3.7. KLF5 Promoted the Proliferation and Invasion of PSCC Cells In Vitro

*KLF5*, an oncogene and a transcriptional activator located in chromosome 13q22.1, has been reported as an HPV integration hotspot gene in other HPV-related cancers [8]. Here, *KLF5* was found to harbor a higher frequency and more support reads of integration breakpoints in PSCC (Supplementary Table S10) than in other cancers. KLF5 expression was upregulated in *KLF5*-integrated tumors (Figure 7a). The carcinogenic role of KLF5 was verified in two PSCC cell lines (Penl2 and 149RCa) in vitro: The knockdown of *KLF5* (Supplementary Figure S9) significantly inhibited the proliferation (*p* < 0.01 and *p* < 0.001; Figure 7b) and invasion of PSCC cells (all *p* < 0.001; Figure 7d). These findings suggest that HPV integration might promote carcinogenesis by enhancing KLF5 expression.

Because KLF5 levels were also higher in tumors with other integration (not KLF5) than that in HPV-negative tumors (*p* <0.001) (Figure 7a), we supposed that HPV integration could also upregulate KLF5 expression in an indirect way. Considering that KLF5 has been reported as a downstream target of the MAPK pathway in colorectal cancer [27], we analyzed the relationship between KLF5 expression and activation of the MAPK pathway, and found that KLF5 expression levels were significantly linearly correlated with the levels of p-ERK (*r* = 0.36, *p* < 0.001), p-JNK (*r* = 0.81, *p* < 0.001), and p-p38 (*r* = 0.75, *p* < 0.001) (Figure 7d). These results suggest that HPV integration may promote KLF5 expression also by activating MAPK pathway.

## 4. Discussion

In the present study, we determined the pattern of HPV integration in PSCC and investigated the impact of HPV integration on penile carcinogenesis. Several findings may contribute a lot to our current knowledge of HPV-induced PSCCs.

First, we identified a carcinogenic mechanism in PSCC that is uncommon in cervical cancer. Significantly fewer integration breakpoints in HPV *E2* were observed than expected in PSCC, whereas no such tendency exists in cervical cancer [9]. Eleven of the 35 (31.4%) PSCCs harbored intact HPV *E2*, indicating that these tumors are not triggered by HPV *E2* gene disruption. Even in 24 tumors with disrupted *E2*, half (12/24) of them only harbored less than three E2 integration sites, suggesting that *E2* disruption may not play a critical role in those tumors. The similar pattern was also observed in other HPV-related squamous cell carcinomas, such as oropharynx squamous cell carcinoma [28,29,30]. Moreover, we found that tumors with intact *E2* harbor lower expression of HPV *E6* and *E7*, and higher expression of p53 and pRb than tumors integrating HPV *E2* DNA. These results remind us that many HPV-induced PSCCs are highly possibly generated by directly affecting critical host genes. Meanwhile, patients with intact *E2* had marginally better survival than those with disrupted *E2* (*p* = 0.094). A similar result also exists in oropharynx squamous cell carcinoma [30]. The reason for this survival difference deserves further investigation.

Second, we first depicted the integration pattern of HPV in PSCC in a direct way. Previous studies in other HPV-related cancers have revealed that HPV integration events are more likely to occur inside or near gene regions and in within genome instability elements. Loci at 13q22.1, 8q24.21, 3q28, 17q23.1, and 17q23.2 are the top five hotspot cytoband regions. Specifically, integration-affected hotspot genes include *KLF5* and *KLF12* (13q22.1), *MYC* (8q24.21), and *TP63* and *TPRG1* (3q28) [8]. In PSCC, Busso-Lopes et al. [10] found that copy number variations (CNVs) of 19 genomic regions are commonly detected in HPV^+^ tumors, including in chromosomal regions 2q33.2-q33.3 and 2q35. Macedo et al. [13] also found several loci, such as 2p12-p11.2, 14q32.33, and 2p16.3, presented CNV in most of HPV+ PSCCs, and several CNVs were correlated with clinicopathological factors. However, none of these results could directly indicate whether these differences between HPV-positive and HPV-negative PSCCs were induced by virus. Furthermore, none of these regions have ever been described as HPV integration hotspots in other HPV-related cancers. In this study, we directly defined the landscape of HPV integration in PSCC by HIVID. The HPV integration pattern of PSCC was found to be more similar to that of other HPV-related cancers. For instance, integration events often occurred inside genes (especially intronic) rather than in intergenic regions; even in intergenic regions, breakpoints were more prone to occur near genes (<100 kb). Several top integrated cytobands such as 13q22.1 and 8q24.21 have previously been reported in other cancers. However, some hotspot regions such as 19p13.11, 8p12, and 3q29 have been identified for the first time in the present study of PSCC. Investigation of these regions may enhance our understanding of HPV-related PSCC.

Third, we identified many hotspot genes and pathways that were associated with HPV-induced carcinogenesis in PSCC. One of the hotspot genes was *CADM2*, which has never been reported to be hotspot gene in other HPV-related tumors. All of the integrations in the *CADM2* gene were caused by the HPV16 subtype in our study. *CADM2* has been reported to be a tumor suppressor in hepatocellular carcinoma and in glioma [31,32], and has been shown to inhibit the migration and invasion of tumor cells via the FAK/AKT signaling pathway [31]. In this study, we found that HPV integration may downregulate the expression of CADM2, which subsequently leads to the inhibition of proliferation, migration, and invasion of PSCC cells in vitro. Integration-affected host genes were enriched in several cancer-related pathways, including the MAPK and JAK/STAT signaling pathways. We verified that activation of the MAPK and JAK/STAT signaling pathways were correlated with HPV integration. This result is also partially supported by previous reports, which found genomic alterations of MAPK, JAK/STAT, and other carcinogenic signaling pathways in PSCC [23,33,34,35,36,37]. Additionally, the enrichment of these pathways was more obvious in tumors of a higher histologic grade compared to tumors of a lower grade, suggesting that these pathways may play crucial roles in tumorigenesis and progression of PSCC. Intriguingly, the expression of KLF5, another integration hotspot gene, was positively correlated with the activation of the MAPK pathway, as indicated by the phosphorylation of the ERK, JNK, and p38 proteins, which is supported by a study reporting that KLF5 is a downstream target of the MAPK pathway [27]. Thus, HPV integration may correlate with penile carcinogenesis or PSCC progression by directly regulating KLF5 and the MAPK pathway or the MAPK–KLF5 axis. Moreover, besides the MAPK signaling pathway, the JAK/STAT signaling pathway may also be associated with HPV-driven PSCC carcinogenesis. Similar results have also been reported in cervical cancer [38].

The major limitation of this study is the relatively small sample size (35 integrated tumors), which limits making a definite conclusion. As PSCC is a rare tumor, a large-scale study would be difficult to carry out. In addition to the inactivation of the tumor-suppressor *CADM2* gene and the activation of the oncogene *KLF5*, as well as the MAPK and JAK/STAT signaling pathways, other effects induced by HPV integration may also exist. As several new HPV integration clustered regions (such as 19p13.11, 8p12, and 3q29) were observed in PSCC, we plan to investigate hotspot genes in these regions to further clarify the additional carcinogenic mechanisms involved in PSCC.

## 5. Conclusions

We described a whole genome-wide study of HPV integration in PSCC, and identified an HPV-related carcinogenic mechanism not only through the traditional *E6*/*E7* carcinogenic process, but also characterized by affecting the host genome, which resulted in the inactivation of tumor suppressors (*CADM2*, etc.) and the activation of oncogenes (*KLF5*, etc.) and oncogenic signaling pathways (MAPK, JAK/STAT, etc.). Our findings enhance our understanding of HPV integration and pave the way for subsequent HPV studies in PSCC. Further large-scale multicenter studies and mechanical studies of *KLF5* and *CADM2* are warranted.

## Figures and Tables

**Table 1:** Association between clinicopathologic characteristics and HPV integration in patients with PSCC.

Variables		No. of Cases	No. of HPV-Negative Tumors (%)	No. of HPV-Positive Tumors with Integration (%)	*p*-Value ^2^	Median Integrations in Positive Tumors	*p*-Value ^3^
Total		102	67 (65.7)	35 (34.3)			
Age (years)	<55 ≧55	48 54	31 (64.6) 36 (66.7)	17 (35.4) 18 (33.3)	0.825	14.0 15.0	0.613
Phimosis	No Yes	44 58	29 (65.9) 38 (65.5)	15 (34.1) 20 (34.5)	0.967	14.0 14.0	0.705
Smoking history	No Yes	38 64	22 (57.9) 45 (70.3)	16 (42.1) 19 (39.7)	0.266	30.0 9.0	0.002
T stage	T_1_ T_2_ or higher	40 62	27 (67.5) 40 (64.5)	13 (32.5) 22 (35.5)	0.757	14.0 14.5	0.960
N stage	N_0_ N_+_	52 50	38 (73.1) 29 (58.0)	14 (26.9) 21 (42.0)	0.109	13.5 15.0	0.987
Histologic grade	G_1_ G_2_ G_3_	48 38 16	38 (79.2) 22 (57.9) 7 (43.8)	10 (20.8) 16 (42.1) 9 (56.2)	0.016	10.0 14.5 46.0	0.105
Histologic subtype ^1^	Non-HPV-related HPV-related	79 23	62 (78.5) 5 (21.7)	17 (21.5) 18 (78.3)	<0.001	14.0 15.0	0.732

^1^ Non-HPV-related PSCC, including usual type, verrucous, papillary, sarcomatoid, and adenosquamous SCC; HPV-related PSCC, including warty, basaloid, and clear-cell SCC. *p*-Values were calculated by ^2^ two-tailed Pearson’s chi-square analysis and ^3^ two-tailed Mann–Whitney or Kruskal–Wallis analysis. FFPE, formalin-fixed, paraffin-embedded.

## Data Availability

The HIVID data generated in this study are available in the NCBI Sequence Read Archive (SRA) under accession numbers SRR9112949–SRR9112989. The other data that support the findings of this study are available from the corresponding author upon request.

## Supplementary Materials

The following are available online at https://www.mdpi.com/article/10.3390/cancers13236104/s1, Figure S1: The pipeline of bioinformatic analysis, Figure S2: Integrated HPV subtypes and clinicopathological/epidemiological characteristics of 35 PSCC samples, Figure S3: The distribution of integration breakpoints in human chromosomes, Figure S4: The integration breakpoints in genomes of the HPV 16 and other HPV subtypes, Figure S5: Families of integration-affected genes, Figure S6: The activation status of Wnt signaling pathways in PSCC specimens, Figure S7: The expression of integration hotspot genes in PSCC, Figure S8: The expression levels of CADM2 in PSCC cell lines, Figure S9: The expression levels of KLF5 in PSCC cell lines, Table S1: High-throughput viral integration detection probes of all 18 HPV types, Table S2: Quality statistics of HIVID-detected samples, Table S3: NCBI accession numbers for HPV, Table S4: Total number of reads uniquely mapped to detected HPV type, Table S5: The information of siRNAs used in this study, Table S6: The primers for quantitative real-time PCR, Table S7: HPV subtypes identified by high-throughput viral integration detection, Table S8: Summary of HPV integration in 35 PSCC samples, Table S9: Integration breakpoints of all HPV subtypes in PSCC, Table S10: Genes affected by HPV integrations detected from virus capture sequencing data, Table S11: Families of HPV integration-affected genes, Table S12: KEGG analysis of integration-related human genes.
