# Supplementary material for: Genome-Wide Profiling Reveals HPV Integration Pattern and Activated Carcinogenic Pathways in Penile Squamous Cell Carcinoma
Source: Cancers (Basel). 2021 Dec 3;13(23):6104. doi: 10.3390/cancers13236104 (PMC8657281; doi:10.3390/cancers13236104)
Supplement: Supplementary file 1 [file cancers-13-06104-s001.zip › Supplementary Figures.pdf]

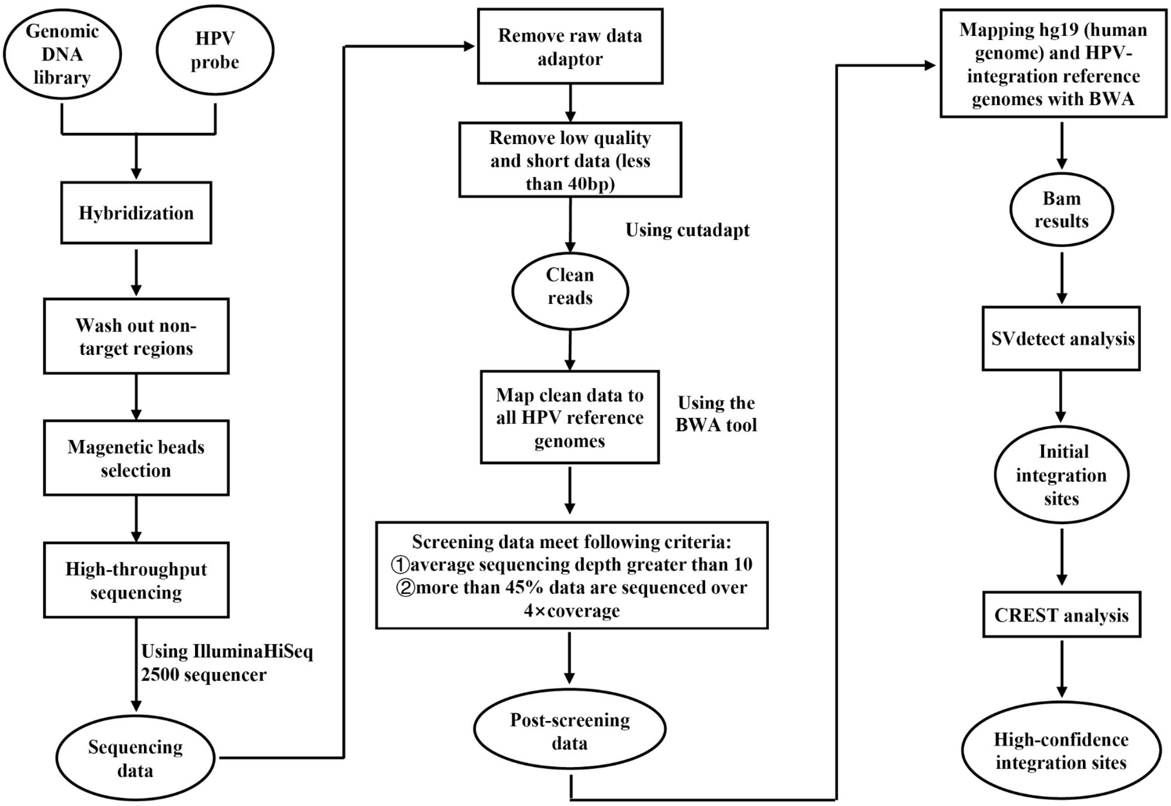

Supplementary Figure S1 - The pipeline of bioinformatic analysis. The bioinformatic process from high-throughput viral integration detection to the identification of integration breakpoints.

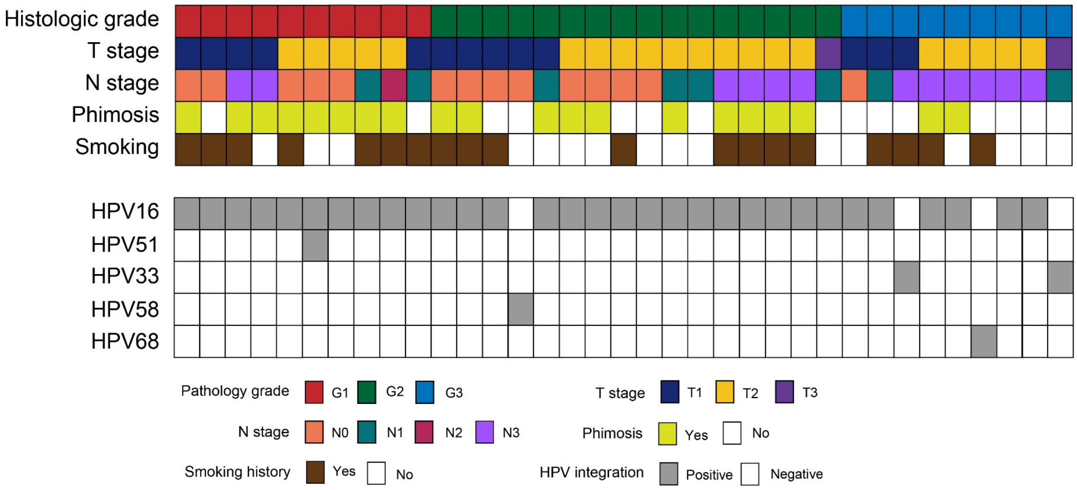

Supplementary Figure S2 - Integrated HPV subtypes and clinicopathological/ epidemiological characteristics of 35 PSCC samples. Each vertical track represents an individual. Top: The main clinicopathological/epidemiological data are sorted by histologic grade; bottom: HPV integration status of different HPV subtypes in patients.

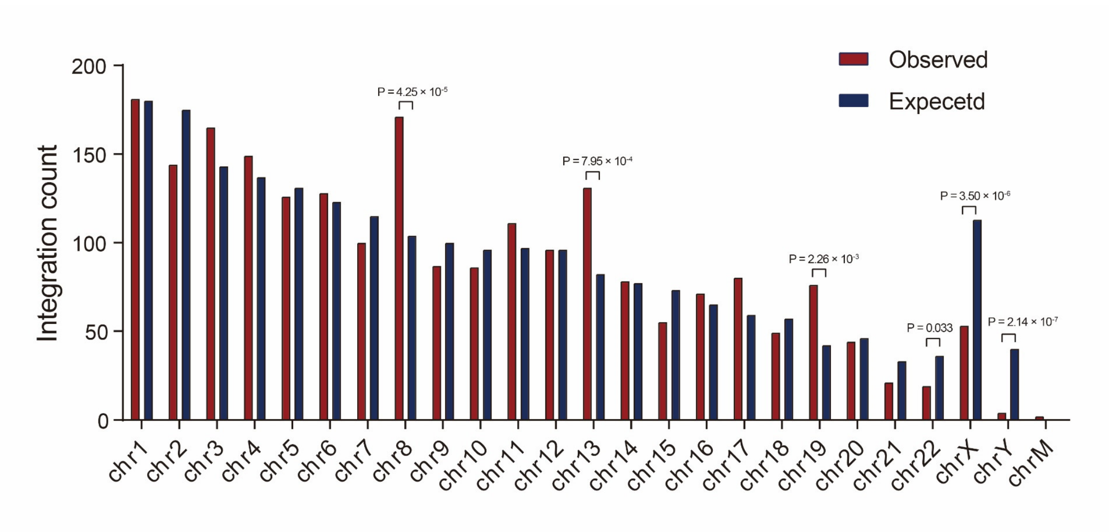

Supplementary Figure S3 - The distribution of integration breakpoints in human chromosomes. Observed (red) and expected (blue) numbers of breakpoints in human chromosomes were compared. Chr, chromosome. *P* values were calculated by the chi-squared tests.

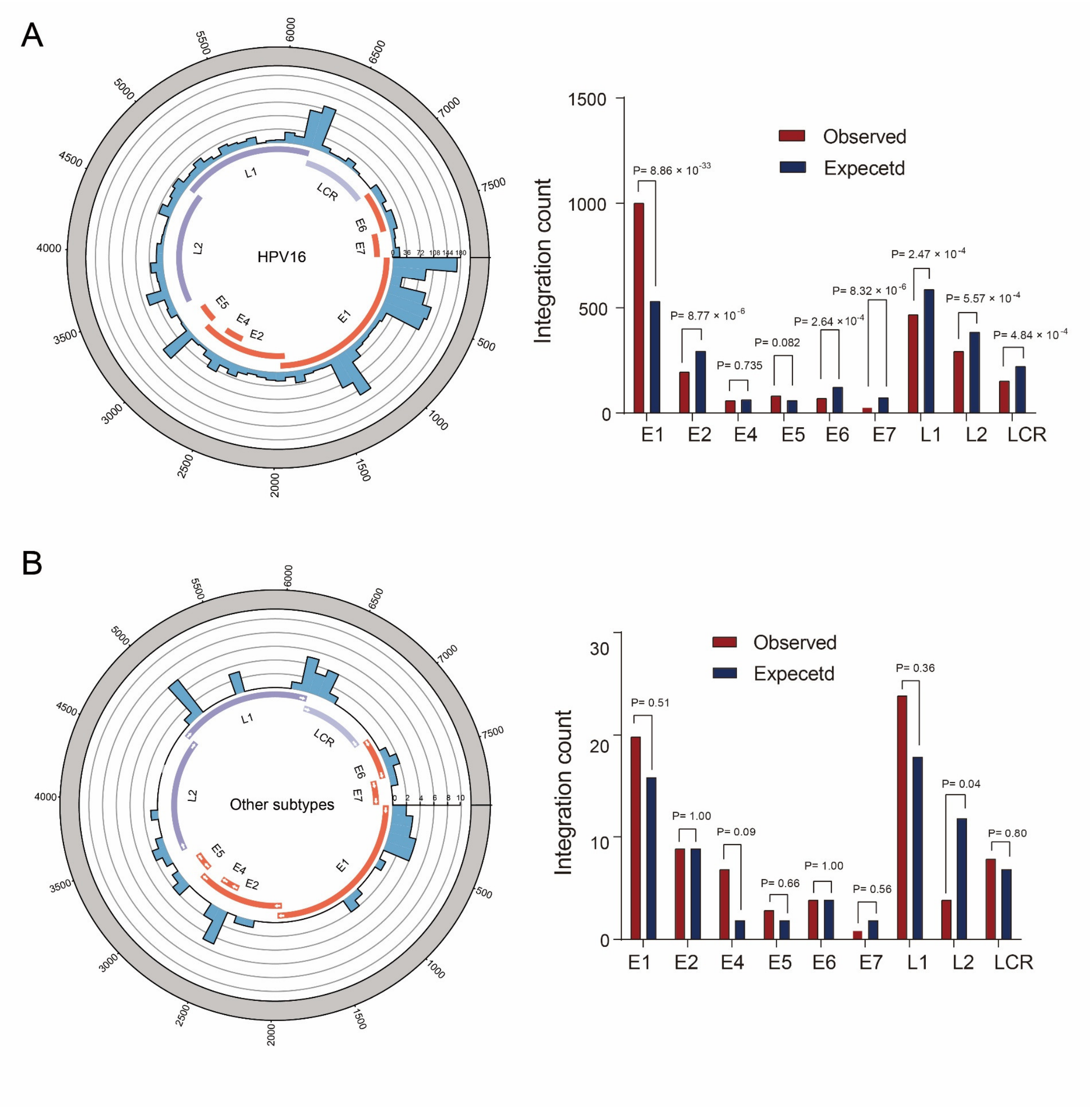

Supplementary Figure S4 - The integration breakpoints in genomes of the HPV 16 and other HPV subtypes. (A) The distribution of integration breakpoints in the HPV16 genome. **(B)** The distribution of integration breakpoints in the genomes of other HPV subtypes. Observed (red) and expected (blue) numbers of breakpoints were compared. *P* values were calculated by the chi-squared tests.

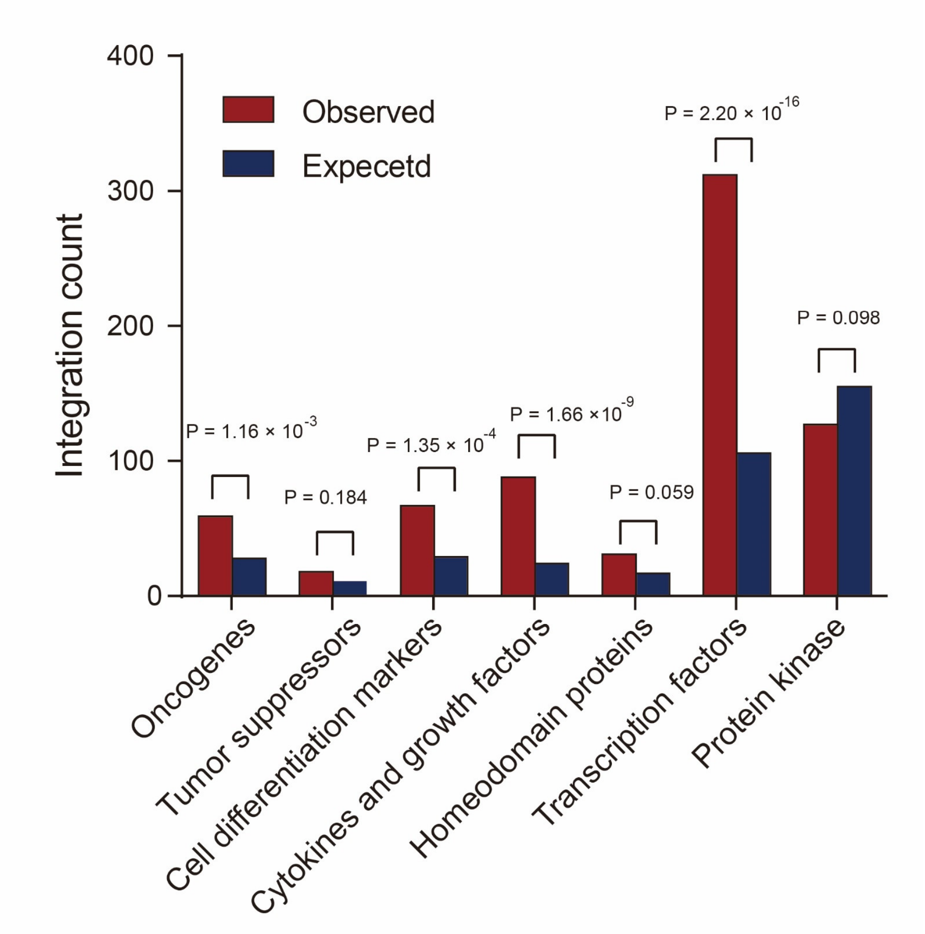

Supplementary Figure S5 - Families of integration-affected genes. Integration-affected genes were classified into different families by Gene Set Enrichment Analysis. *P* values were calculated by the chi-squared tests.

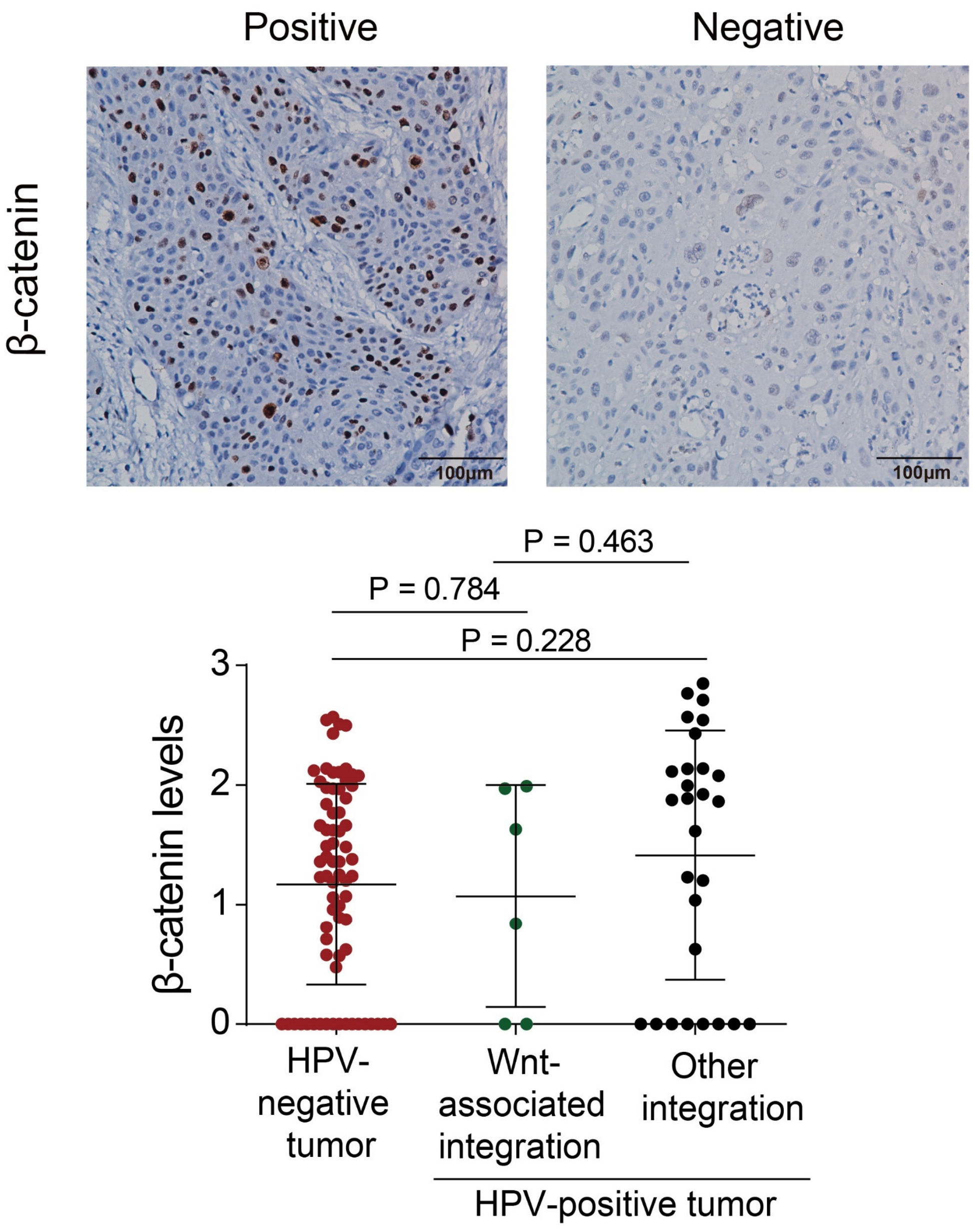

Supplementary Figure S6 - The activation status of Wnt signaling pathways in PSCC specimens. The activation status of Wnt signaling pathway was evaluated by the expression of nucleic β-catenin. Upper, Representative IHC images (100×); Lower, bar chart for the levels of these factors in HPV-negative tumors, HPV-positive tumors with MAPK-associated integration and HPV-positive tumors with other integration. Statistical significance was set as $P < 0.05$.

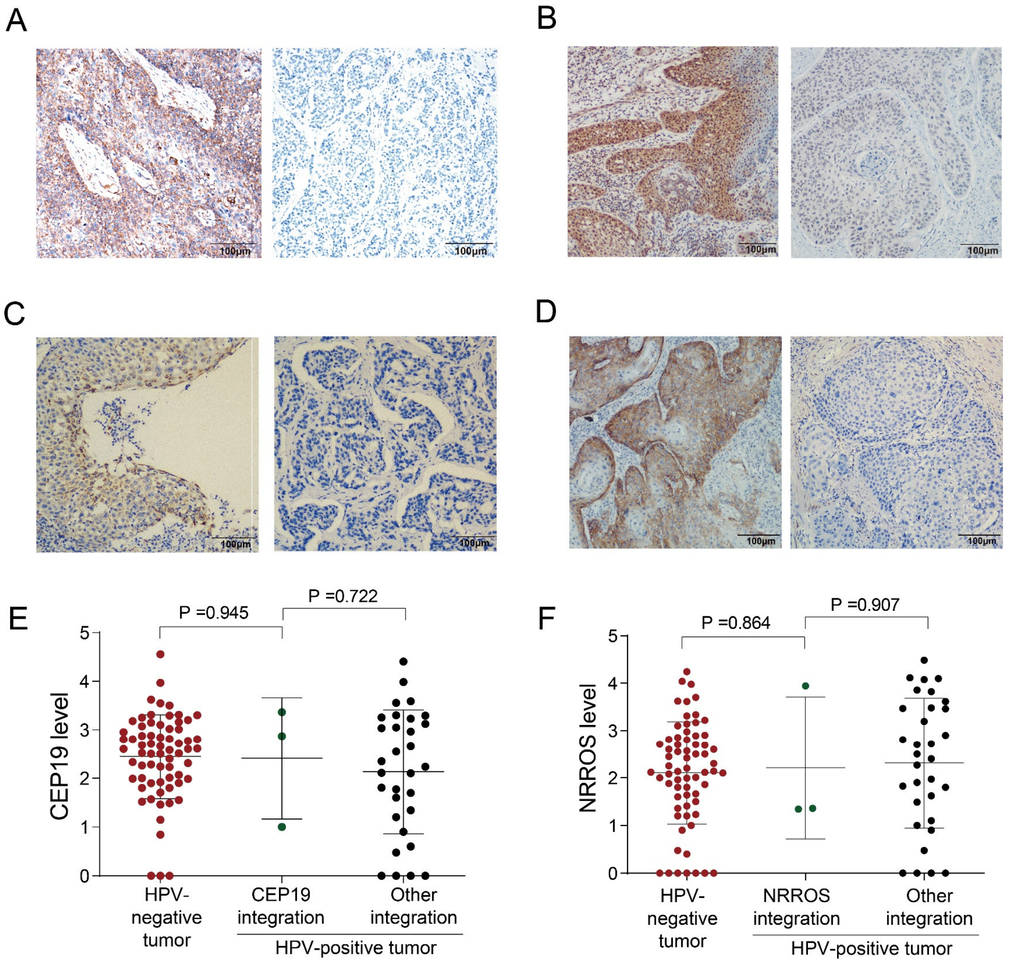

Supplementary Figure S7 - The expression of integration hotspot genes in PSCC. (**A-D**) Representative IHC images (100×) for CADM2 (A), KLF5 (B), CEP19 (C) and NRROS (D) expression (left: positive sample; right: negative sample); (**E**) CEP19 expression, represented by log_2(integrated optical density + 1) value, in HPV-negative tumors, HPV-positive tumors with CEP19 integration and HPV-positive tumors with other integration. (**F**) NRROS expression in HPV-negative tumors, HPV-positive tumors with NRROS integration and HPV-positive tumors with other integration. The significance of the difference was analyzed by ANOVA. Statistical significance was set as $P < 0.05$.

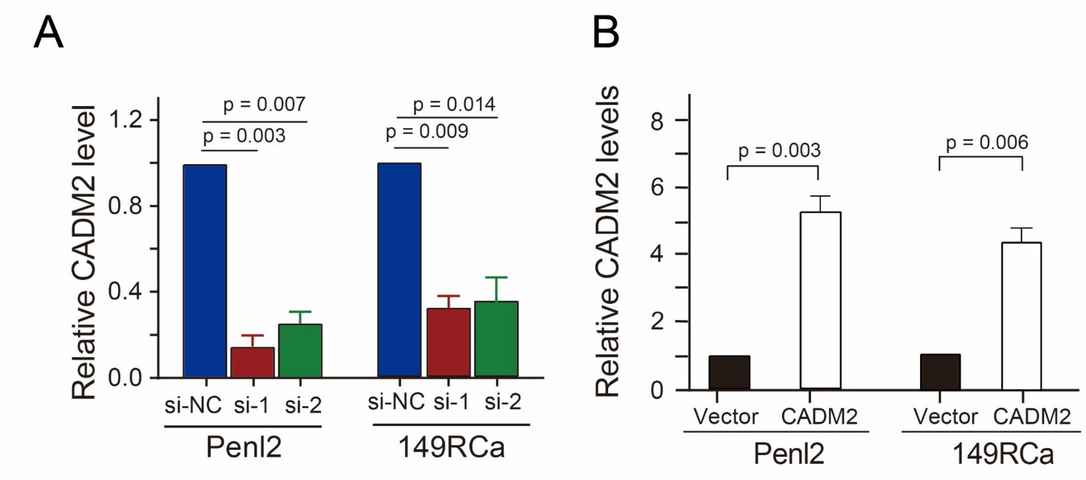

Supplementary Figure S8 - The expression levels of CADM2 in PSCC cell lines. Relative CADM2 levels in PSCC cell lines with CADM2-knockdown (**A**) or CADM2-overexpression (**B**), examined by qPCR analysis. *P* value was calculated via Student's *t* test.

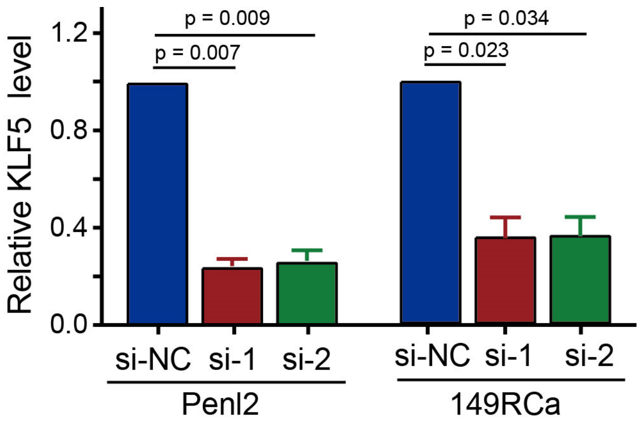

Supplementary Figure S9 - The expression levels of KLF5 in PSCC cell lines. Relative KLF5 levels in PSCC cell lines with KLF5-knockdown or KLF5-overexpression, examined by qPCR assay. *P* value was calculated via Student's *t* test.
